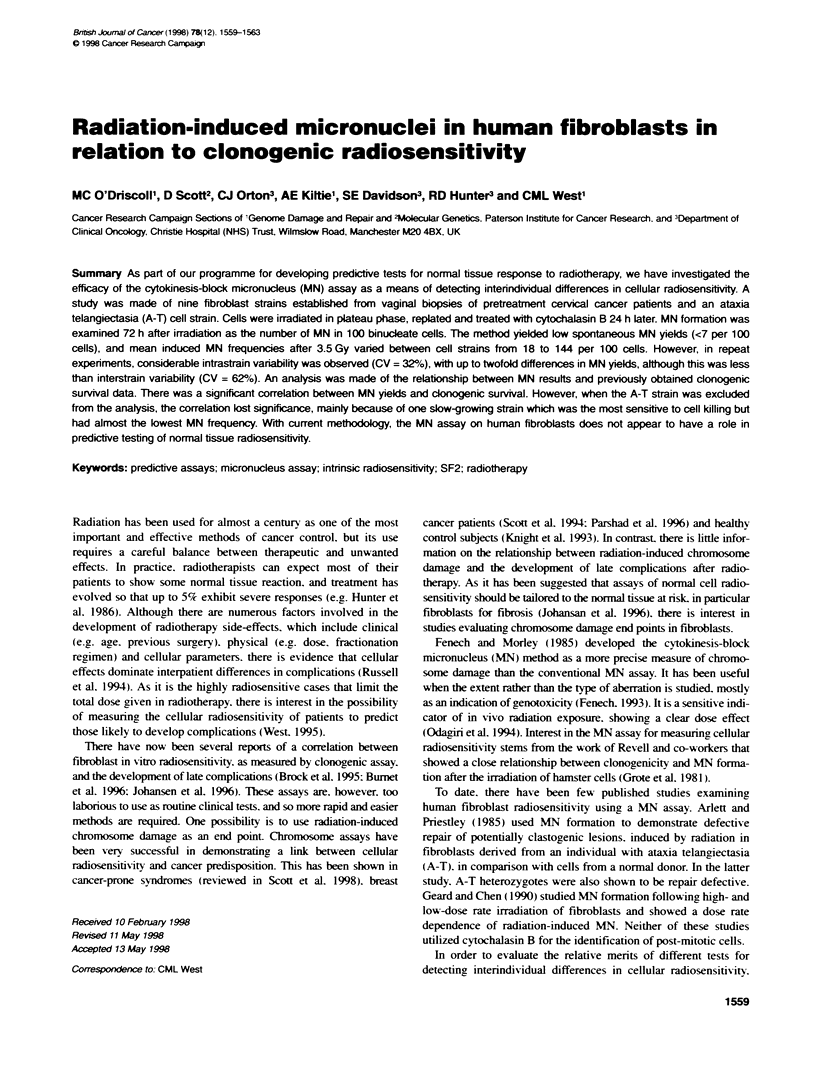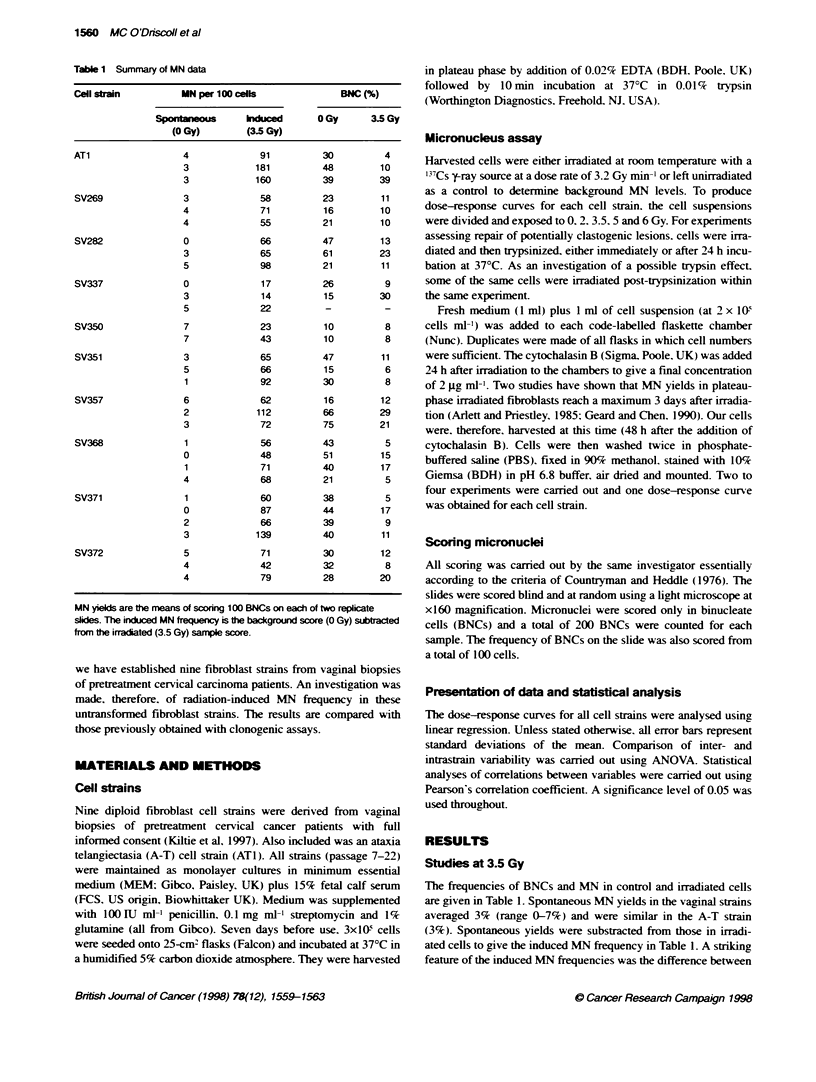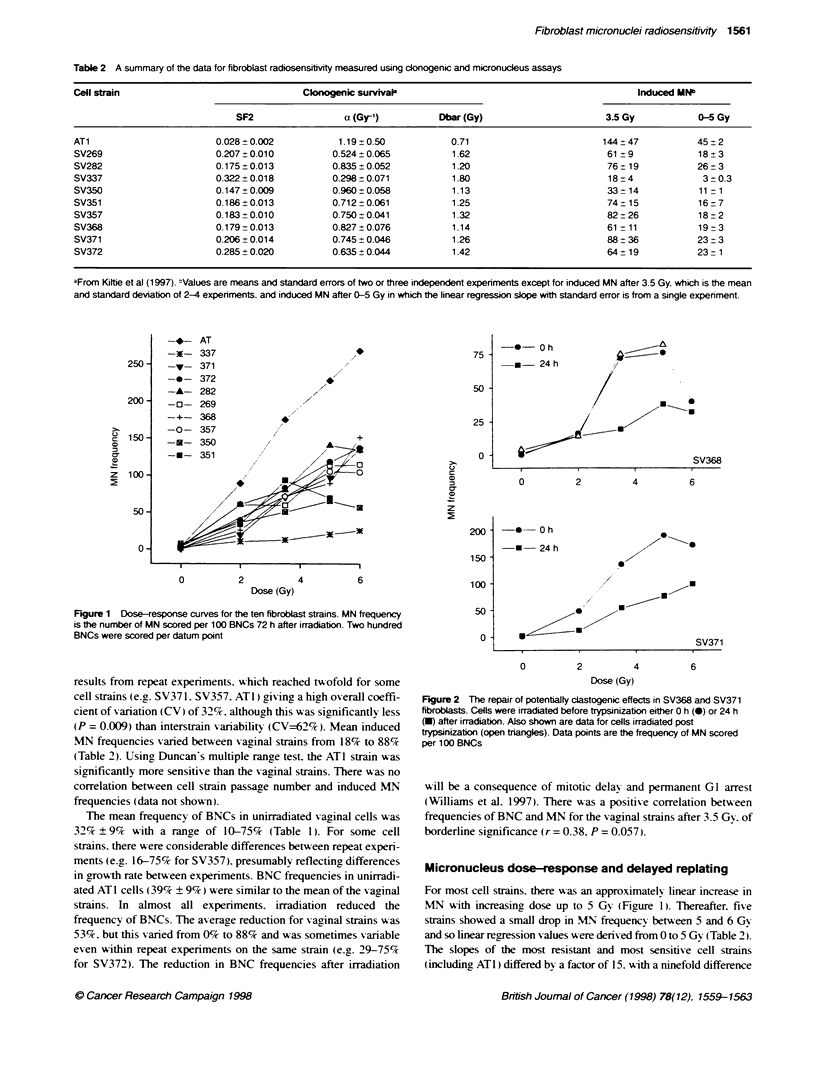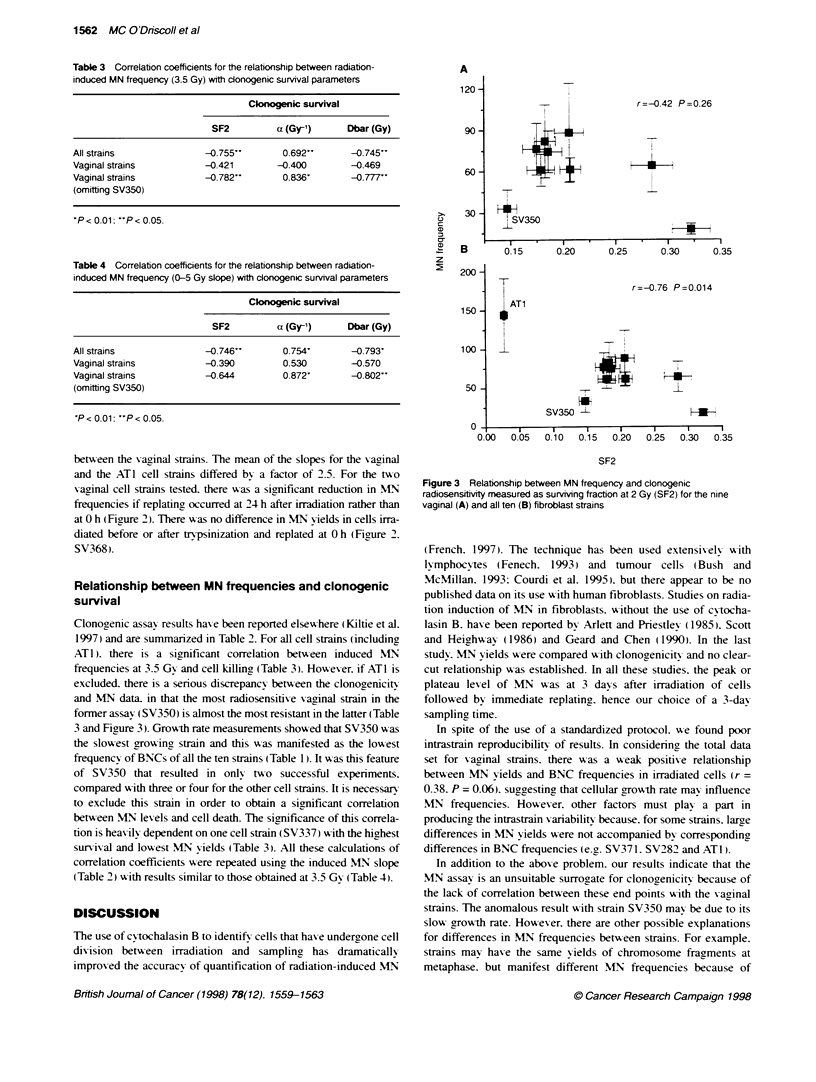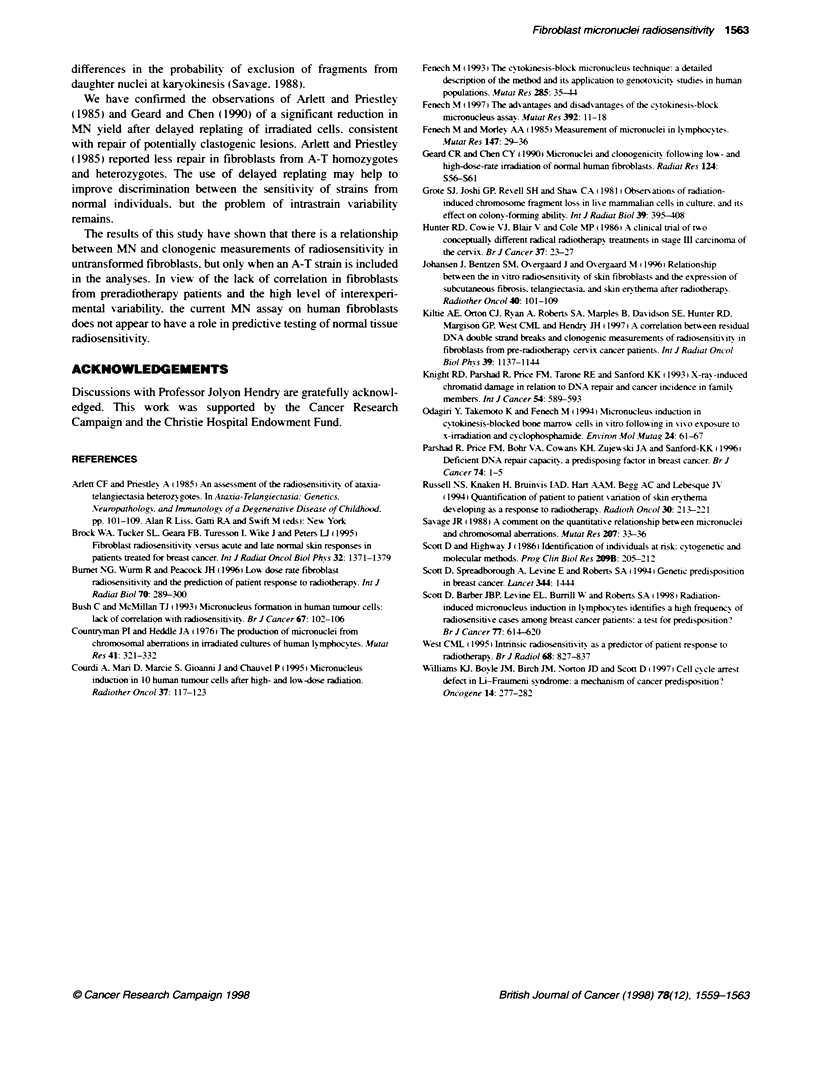# Radiation-induced micronuclei in human fibroblasts in relation to clonogenic radiosensitivity.

**DOI:** 10.1038/bjc.1998.723

**Published:** 1998-12

**Authors:** M. C. O'Driscoll, D. Scott, C. J. Orton, A. E. Kiltie, S. E. Davidson, R. D. Hunter, C. M. West

**Affiliations:** Cancer Research Campaign Section of Genome Damage and Repair, Paterson Institute for Cancer Research, Christie Hospital (NHS) Trust, Manchester, UK.

## Abstract

As part of our programme for developing predictive tests for normal tissue response to radiotherapy, we have investigated the efficacy of the cytokinesis-block micronucleus (MN) assay as a means of detecting interindividual differences in cellular radiosensitivity. A study was made of nine fibroblast strains established from vaginal biopsies of pretreatment cervical cancer patients and an ataxia telangiectasia (A-T) cell strain. Cells were irradiated in plateau phase, replated and treated with cytochalasin B 24 h later. MN formation was examined 72 h after irradiation as the number of MN in 100 binucleate cells. The method yielded low spontaneous MN yields (<7 per 100 cells), and mean induced MN frequencies after 3.5 Gy varied between cell strains from 18 to 144 per 100 cells. However, in repeat experiments, considerable intrastrain variability was observed (CV = 32%), with up to twofold differences in MN yields, although this was less than interstrain variability (CV = 62%). An analysis was made of the relationship between MN results and previously obtained clonogenic survival data. There was a significant correlation between MN yields and clonogenic survival. However, when the A-T strain was excluded from the analysis, the correlation lost significance, mainly because of one slow-growing strain which was the most sensitive to cell killing but had almost the lowest MN frequency. With current methodology, the MN assay on human fibroblasts does not appear to have a role in predictive testing of normal tissue radiosensitivity.


British Journal of Cancer (1998) 78(12). 1559–1563
© 1998 Cancer Research Campaign

### Radiation-induced micronuclei in human fibroblasts in relation to clonogenic radiosensitivity

**MC O'Driscoll[1], D Scott[2], CJ Orton[3], AE Kiltie[1], SE Davidson[3], RD Hunter[3] and CML West[1]**

Cancer Research Campaign Sections of [1]Genome Damage and Repair and [2]Molecular Genetics, Paterson Institute for Cancer Research, and [3]Department of Clinical Oncology, Christie Hospital (NHS) Trust, Wilmslow Road, Manchester M20 4BX, UK

**Summary** As part of our programme for developing predictive tests for normal tissue response to radiotherapy, we have investigated the efficacy of the cytokinesis-block micronucleus (MN) assay as a means of detecting interindividual differences in cellular radiosensitivity. A study was made of nine fibroblast strains established from vaginal biopsies of pretreatment cervical cancer patients and an ataxia telangiectasia (A-T) cell strain. Cells were irradiated in plateau phase, replated and treated with cytochalasin B 24 h later. MN formation was examined 72 h after irradiation as the number of MN in 100 binucleate cells. The method yielded low spontaneous MN yields (<7 per 100 cells), and mean induced MN frequencies after 3.5 Gy varied between cell strains from 18 to 144 per 100 cells. However, in repeat experiments, considerable intrastrain variability was observed (CV = 32%), with up to twofold differences in MN yields, although this was less than interstrain variability (CV = 62%). An analysis was made of the relationship between MN results and previously obtained clonogenic survival data. There was a significant correlation between MN yields and clonogenic survival. However, when the A-T strain was excluded from the analysis, the correlation lost significance, mainly because of one slow-growing strain which was the most sensitive to cell killing but had almost the lowest MN frequency. With current methodology, the MN assay on human fibroblasts does not appear to have a role in predictive testing of normal tissue radiosensitivity.

**Keywords:** predictive assays; micronucleus assay; intrinsic radiosensitivity; SF2; radiotherapy

Radiation has been used for almost a century as one of the most important and effective methods of cancer control, but its use requires a careful balance between therapeutic and unwanted effects. In practice, radiotherapists can expect most of their patients to show some normal tissue reaction, and treatment has evolved so that up to 5% exhibit severe responses (e.g. Hunter et al. 1986). Although there are numerous factors involved in the development of radiotherapy side-effects, which include clinical (e.g. age, previous surgery), physical (e.g. dose, fractionation regimen) and cellular parameters, there is evidence that cellular effects dominate interpatient differences in complications (Russell et al. 1994). As it is the highly radiosensitive cases that limit the total dose given in radiotherapy, there is interest in the possibility of measuring the cellular radiosensitivity of patients to predict those likely to develop complications (West, 1995).

There have now been several reports of a correlation between fibroblast in vitro radiosensitivity, as measured by clonogenic assay, and the development of late complications (Brock et al. 1995; Burnet et al. 1996; Johansen et al. 1996). These assays are, however, too laborious to use as routine clinical tests, and so more rapid and easier methods are required. One possibility is to use radiation-induced chromosome damage as an end point. Chromosome assays have been very successful in demonstrating a link between cellular radiosensitivity and cancer predisposition. This has been shown in cancer-prone syndromes (reviewed in Scott et al. 1998), breast cancer patients (Scott et al. 1994; Parshad et al. 1996) and healthy control subjects (Knight et al. 1993). In contrast, there is little information on the relationship between radiation-induced chromosome damage and the development of late complications after radiotherapy. As it has been suggested that assays of normal cell radiosensitivity should be tailored to the normal tissue at risk, in particular fibroblasts for fibrosis (Johansan et al. 1996), there is interest in studies evaluating chromosome damage end points in fibroblasts.

Fenech and Morley (1985) developed the cytokinesis-block micronucleus (MN) method as a more precise measure of chromosome damage than the conventional MN assay. It has been useful when the extent rather than the type of aberration is studied, mostly as an indication of genotoxicity (Fenech, 1993). It is a sensitive indicator of in vivo radiation exposure, showing a clear dose effect (Odagiri et al. 1994). Interest in the MN assay for measuring cellular radiosensitivity stems from the work of Revell and co-workers that showed a close relationship between clonogenicity and MN formation after the irradiation of hamster cells (Grote et al. 1981).

To date, there have been few published studies examining human fibroblast radiosensitivity using a MN assay. Arlett and Priestley (1985) used MN formation to demonstrate defective repair of potentially clastogenic lesions, induced by radiation in fibroblasts derived from an individual with ataxia telangiectasia (A-T), in comparison with cells from a normal donor. In the latter study, A-T heterozygotes were also shown to be repair defective. Geard and Chen (1990) studied MN formation following high- and low-dose rate irradiation of fibroblasts and showed a dose rate dependence of radiation-induced MN. Neither of these studies utilized cytochalasin B for the identification of post-mitotic cells.

In order to evaluate the relative merits of different tests for detecting interindividual differences in cellular radiosensitivity,

*Received 10 February 1998*
*Revised 11 May 1998*
*Accepted 13 May 1998*

*Correspondence to:* CML West

Table 1 Summary of MN data

| Cell strain | MN per 100 cells | | BNC (%) | |
|---|---|---|---|---|
| | Spontaneous (0 Gy) | Induced (3.5 Gy) | 0 Gy | 3.5 Gy |
| AT1 | 4 | 91 | 30 | 4 |
| | 3 | 181 | 48 | 10 |
| | 3 | 160 | 39 | 39 |
| SV269 | 3 | 58 | 23 | 11 |
| | 4 | 71 | 16 | 10 |
| | 4 | 55 | 21 | 10 |
| SV282 | 0 | 66 | 47 | 13 |
| | 3 | 65 | 61 | 23 |
| | 5 | 98 | 21 | 11 |
| SV337 | 0 | 17 | 26 | 9 |
| | 3 | 14 | 15 | 30 |
| | 5 | 22 | – | – |
| SV350 | 7 | 23 | 10 | 8 |
| | 7 | 43 | 10 | 8 |
| SV351 | 3 | 65 | 47 | 11 |
| | 5 | 66 | 15 | 6 |
| | 1 | 92 | 30 | 8 |
| SV357 | 6 | 62 | 16 | 12 |
| | 2 | 112 | 66 | 29 |
| | 3 | 72 | 75 | 21 |
| SV368 | 1 | 56 | 43 | 5 |
| | 0 | 48 | 51 | 15 |
| | 1 | 71 | 40 | 17 |
| | 4 | 68 | 21 | 5 |
| SV371 | 1 | 60 | 38 | 5 |
| | 0 | 87 | 44 | 17 |
| | 2 | 66 | 39 | 9 |
| | 3 | 139 | 40 | 11 |
| SV372 | 5 | 71 | 30 | 12 |
| | 4 | 42 | 32 | 8 |
| | 4 | 79 | 28 | 20 |

MN yields are the means of scoring 100 BNCs on each of two replicate slides. The induced MN frequency is the background score (0 Gy) subtracted from the irradiated (3.5 Gy) sample score.

we have established nine fibroblast strains from vaginal biopsies of pretreatment cervical carcinoma patients. An investigation was made, therefore, of radiation-induced MN frequency in these untransformed fibroblast strains. The results are compared with those previously obtained with clonogenic assays.

### MATERIALS AND METHODS

### Cell strains

Nine diploid fibroblast cell strains were derived from vaginal biopsies of pretreatment cervical cancer patients with full informed consent (Kiltie et al, 1997). Also included was an ataxia telangiectasia (A-T) cell strain (AT1). All strains (passage 7–22) were maintained as monolayer cultures in minimum essential medium (MEM; Gibco, Paisley, UK) plus 15% fetal calf serum (FCS, US origin, Biowhittaker UK). Medium was supplemented with 100 IU ml$^{-1}$ penicillin, 0.1 mg ml$^{-1}$ streptomycin and 1% glutamine (all from Gibco). Seven days before use, $3\times10^5$ cells were seeded onto 25-cm$^2$ flasks (Falcon) and incubated at 37°C in a humidified 5% carbon dioxide atmosphere. They were harvested in plateau phase by addition of 0.02% EDTA (BDH, Poole, UK) followed by 10 min incubation at 37°C in 0.01% trypsin (Worthington Diagnostics, Freehold, NJ, USA).

### Micronucleus assay

Harvested cells were either irradiated at room temperature with a $^{137}Cs$ γ-ray source at a dose rate of 3.2 Gy min$^{-1}$ or left unirradiated as a control to determine background MN levels. To produce dose–response curves for each cell strain, the cell suspensions were divided and exposed to 0, 2, 3.5, 5 and 6 Gy. For experiments assessing repair of potentially clastogenic lesions, cells were irradiated and then trypsinized, either immediately or after 24 h incubation at 37°C. As an investigation of a possible trypsin effect, some of the same cells were irradiated post-trypsinization within the same experiment.

Fresh medium (1 ml) plus 1 ml of cell suspension (at $2\times10^5$ cells ml$^{-1}$) was added to each code-labelled flaskette chamber (Nunc). Duplicates were made of all flasks in which cell numbers were sufficient. The cytochalasin B (Sigma, Poole, UK) was added 24 h after irradiation to the chambers to give a final concentration of 2 μg ml$^{-1}$. Two studies have shown that MN yields in plateau-phase irradiated fibroblasts reach a maximum 3 days after irradiation (Arlett and Priestley, 1985; Geard and Chen, 1990). Our cells were, therefore, harvested at this time (48 h after the addition of cytochalasin B). Cells were then washed twice in phosphate-buffered saline (PBS), fixed in 90% methanol, stained with 10% Giemsa (BDH) in pH 6.8 buffer, air dried and mounted. Two to four experiments were carried out and one dose–response curve was obtained for each cell strain.

### Scoring micronuclei

All scoring was carried out by the same investigator essentially according to the criteria of Countryman and Heddle (1976). The slides were scored blind and at random using a light microscope at ×160 magnification. Micronuclei were scored only in binucleate cells (BNCs) and a total of 200 BNCs were counted for each sample. The frequency of BNCs on the slide was also scored from a total of 100 cells.

### Presentation of data and statistical analysis

The dose–response curves for all cell strains were analysed using linear regression. Unless stated otherwise, all error bars represent standard deviations of the mean. Comparison of inter- and intrastrain variability was carried out using ANOVA. Statistical analyses of correlations between variables were carried out using Pearson's correlation coefficient. A significance level of 0.05 was used throughout.

### RESULTS

### Studies at 3.5 Gy

The frequencies of BNCs and MN in control and irradiated cells are given in Table 1. Spontaneous MN yields in the vaginal strains averaged 3% (range 0–7%) and were similar in the A-T strain (3%). Spontaneous yields were substracted from those in irradiated cells to give the induced MN frequency in Table 1. A striking feature of the induced MN frequencies was the difference between

© Cancer Research Campaign 1998

**Table 2** A summary of the data for fibroblast radiosensitivity measured using clonogenic and micronucleus assays

| Cell strain | Clonogenic survival[a] | | | Induced MN[b] | |
|---|---|---|---|---|---|
| | SF2 | α (Gy⁻¹) | Dbar (Gy) | 3.5 Gy | 0–5 Gy |
| AT1 | 0.028 ± 0.002 | 1.19 ± 0.50 | 0.71 | 144 ± 47 | 45 ± 2 |
| SV269 | 0.207 ± 0.010 | 0.524 ± 0.065 | 1.62 | 61 ± 9 | 18 ± 3 |
| SV282 | 0.175 ± 0.013 | 0.835 ± 0.052 | 1.20 | 76 ± 19 | 26 ± 3 |
| SV337 | 0.322 ± 0.018 | 0.298 ± 0.071 | 1.80 | 18 ± 4 | 3 ± 0.3 |
| SV350 | 0.147 ± 0.009 | 0.960 ± 0.058 | 1.13 | 33 ± 14 | 11 ± 1 |
| SV351 | 0.186 ± 0.013 | 0.712 ± 0.061 | 1.25 | 74 ± 15 | 16 ± 7 |
| SV357 | 0.183 ± 0.010 | 0.750 ± 0.041 | 1.32 | 82 ± 26 | 18 ± 2 |
| SV368 | 0.179 ± 0.013 | 0.827 ± 0.076 | 1.14 | 61 ± 11 | 19 ± 3 |
| SV371 | 0.206 ± 0.014 | 0.745 ± 0.046 | 1.26 | 88 ± 36 | 23 ± 3 |
| SV372 | 0.285 ± 0.020 | 0.635 ± 0.044 | 1.42 | 64 ± 19 | 23 ± 1 |

[a]From Kiltie et al (1997). [b]Values are means and standard errors of two or three independent experiments except for induced MN after 3.5 Gy, which is the mean and standard deviation of 2–4 experiments, and induced MN after 0–5 Gy in which the linear regression slope with standard error is from a single experiment.

**Figure 1** Dose–response curves for the ten fibroblast strains. MN frequency is the number of MN scored per 100 BNCs 72 h after irradiation. Two hundred BNCs were scored per datum point

**Figure 2** The repair of potentially clastogenic effects in SV368 and SV371 fibroblasts. Cells were irradiated before trypsinization either 0 h (●) or 24 h (■) after irradiation. Also shown are data for cells irradiated post trypsinization (open triangles). Data points are the frequency of MN scored per 100 BNCs results from repeat experiments, which reached twofold for some cell strains (e.g. SV371, SV357, AT1) giving a high overall coefficient of variation (CV) of 32%, although this was significantly less ($P$ = 0.009) than interstrain variability (CV=62%). Mean induced MN frequencies varied between vaginal strains from 18% to 88% (Table 2). Using Duncan's multiple range test, the AT1 strain was significantly more sensitive than the vaginal strains. There was no correlation between cell strain passage number and induced MN frequencies (data not shown).

The mean frequency of BNCs in unirradiated vaginal cells was 32% ± 9% with a range of 10–75% (Table 1). For some cell strains, there were considerable differences between repeat experiments (e.g. 16–75% for SV357), presumably reflecting differences in growth rate between experiments. BNC frequencies in unirradiated AT1 cells (39% ± 9%) were similar to the mean of the vaginal strains. In almost all experiments, irradiation reduced the frequency of BNCs. The average reduction for vaginal strains was 53%, but this varied from 0% to 88% and was sometimes variable even within repeat experiments on the same strain (e.g. 29–75% for SV372). The reduction in BNC frequencies after irradiation will be a consequence of mitotic delay and permanent G1 arrest (Williams et al, 1997). There was a positive correlation between frequencies of BNC and MN for the vaginal strains after 3.5 Gy, of borderline significance ($r$ = 0.38, $P$ = 0.057).

### Micronucleus dose–response and delayed replating

For most cell strains, there was an approximately linear increase in MN with increasing dose up to 5 Gy (Figure 1). Thereafter, five strains showed a small drop in MN frequency between 5 and 6 Gy and so linear regression values were derived from 0 to 5 Gy (Table 2). The slopes of the most resistant and most sensitive cell strains (including AT1) differed by a factor of 15, with a ninefold difference

Table 3 Correlation coefficients for the relationship between radiation-induced MN frequency (3.5 Gy) with clonogenic survival parameters

| | Clonogenic survival | | |
|---|---|---|---|
| | SF2 | α (Gy⁻¹) | Dbar (Gy) |
| All strains | –0.755** | 0.692** | –0.745** |
| Vaginal strains | –0.421 | –0.400 | –0.469 |
| Vaginal strains (omitting SV350) | –0.782** | 0.836* | –0.777** |

*$P < 0.01$; **$P < 0.05$.

Table 4 Correlation coefficients for the relationship between radiation-induced MN frequency (0–5 Gy slope) with clonogenic survival parameters

| | Clonogenic survival | | |
|---|---|---|---|
| | SF2 | α (Gy⁻¹) | Dbar (Gy) |
| All strains | –0.746** | 0.754* | –0.793* |
| Vaginal strains | –0.390 | 0.530 | –0.570 |
| Vaginal strains (omitting SV350) | –0.644 | 0.872* | –0.802** |

*$P < 0.01$; **$P < 0.05$.

between the vaginal strains. The mean of the slopes for the vaginal and the AT1 cell strains differed by a factor of 2.5. For the two vaginal cell strains tested, there was a significant reduction in MN frequencies if replating occurred at 24 h after irradiation rather than at 0 h (Figure 2). There was no difference in MN yields in cells irradiated before or after trypsinization and replated at 0 h (Figure 2, SV368).

### Relationship between MN frequencies and clonogenic survival

Clonogenic assay results have been reported elsewhere (Kiltie et al, 1997) and are summarized in Table 2. For all cell strains (including AT1), there is a significant correlation between induced MN frequencies at 3.5 Gy and cell killing (Table 3). However, if AT1 is excluded, there is a serious discrepancy between the clonogenicity and MN data, in that the most radiosensitive vaginal strain in the former assay (SV350) is almost the most resistant in the latter (Table 3 and Figure 3). Growth rate measurements showed that SV350 was the slowest growing strain and this was manifested as the lowest frequency of BNCs of all the ten strains (Table 1). It was this feature of SV350 that resulted in only two successful experiments, compared with three or four for the other cell strains. It is necessary to exclude this strain in order to obtain a significant correlation between MN levels and cell death. The significance of this correlation is heavily dependent on one cell strain (SV337) with the highest survival and lowest MN yields (Table 3). All these calculations of correlation coefficients were repeated using the induced MN slope (Table 2) with results similar to those obtained at 3.5 Gy (Table 4).

### DISCUSSION

The use of cytochalasin B to identify cells that have undergone cell division between irradiation and sampling has dramatically improved the accuracy of quantification of radiation-induced MN

Figure 3 Relationship between MN frequency and clonogenic radiosensitivity measured as surviving fraction at 2 Gy (SF2) for the nine vaginal (A) and all ten (B) fibroblast strains

(French, 1997). The technique has been used extensively with lymphocytes (Fenech, 1993) and tumour cells (Bush and McMillan, 1993; Courdi et al, 1995), but there appear to be no published data on its use with human fibroblasts. Studies on radiation induction of MN in fibroblasts, without the use of cytochalasin B, have been reported by Arlett and Priestley (1985), Scott and Heighway (1986) and Geard and Chen (1990). In the last study, MN yields were compared with clonogenicity and no clear-cut relationship was established. In all these studies, the peak or plateau level of MN was at 3 days after irradiation of cells followed by immediate replating, hence our choice of a 3-day sampling time.

In spite of the use of a standardized protocol, we found poor intrastrain reproducibility of results. In considering the total data set for vaginal strains, there was a weak positive relationship between MN yields and BNC frequencies in irradiated cells ($r = 0.38$, $P = 0.06$), suggesting that cellular growth rate may influence MN frequencies. However, other factors must play a part in producing the intrastrain variability because, for some strains, large differences in MN yields were not accompanied by corresponding differences in BNC frequencies (e.g. SV371, SV282 and AT1).

In addition to the above problem, our results indicate that the MN assay is an unsuitable surrogate for clonogenicity because of the lack of correlation between these end points with the vaginal strains. The anomalous result with strain SV350 may be due to its slow growth rate. However, there are other possible explanations for differences in MN frequencies between strains. For example, strains may have the same yields of chromosome fragments at metaphase, but manifest different MN frequencies because of

© Cancer Research Campaign 1998

differences in the probability of exclusion of fragments from daughter nuclei at karyokinesis (Savage, 1988).

We have confirmed the observations of Arlett and Priestley (1985) and Geard and Chen (1990) of a significant reduction in MN yield after delayed replating of irradiated cells, consistent with repair of potentially clastogenic lesions. Arlett and Priestley (1985) reported less repair in fibroblasts from A-T homozygotes and heterozygotes. The use of delayed replating may help to improve discrimination between the sensitivity of strains from normal individuals, but the problem of intrastrain variability remains.

The results of this study have shown that there is a relationship between MN and clonogenic measurements of radiosensitivity in untransformed fibroblasts, but only when an A-T strain is included in the analyses. In view of the lack of correlation in fibroblasts from preradiotherapy patients and the high level of interexperimental variability, the current MN assay on human fibroblasts does not appear to have a role in predictive testing of normal tissue radiosensitivity.

### ACKNOWLEDGEMENTS

Discussions with Professor Jolyon Hendry are gratefully acknowledged. This work was supported by the Cancer Research Campaign and the Christie Hospital Endowment Fund.

### REFERENCES

Arlett CF and Priestley A (1985) An assessment of the radiosensitivity of ataxia-telangiectasia heterozygotes. In *Ataxia-Telangiectasia: Genetics, Neuropathology, and Immunology of a Degenerative Disease of Childhood*. pp. 101–109. Alan R Liss. Gatti RA and Swift M (eds): New York

Brock WA, Tucker SL, Geara FB, Turesson I, Wike J and Peters LJ (1995) Fibroblast radiosensitivity versus acute and late normal skin responses in patients treated for breast cancer. *Int J Radiat Oncol Biol Phys* **32**: 1371–1379

Burnet NG, Wurm R and Peacock JH (1996) Low dose rate fibroblast radiosensitivity and the prediction of patient response to radiotherapy. *Int J Radiat Biol* **70**: 289–300

Bush C and McMillan TJ (1993) Micronucleus formation in human tumour cells: lack of correlation with radiosensitivity. *Br J Cancer* **67**: 102–106

Countryman PI and Heddle JA (1976) The production of micronuclei from chromosomal aberrations in irradiated cultures of human lymphocytes. *Mutat Res* **41**: 321–332

Courdi A, Mari D, Marcie S, Gioanni J and Chauvel P (1995) Micronucleus induction in 10 human tumour cells after high- and low-dose radiation. *Radiother Oncol* **37**: 117–123

Fenech M (1993) The cytokinesis-block micronucleus technique: a detailed description of the method and its application to genotoxicity studies in human populations. *Mutat Res* **285**: 35–44

Fenech M (1997) The advantages and disadvantages of the cytokinesis-block micronucleus assay. *Mutat Res* **392**: 11–18

Fenech M and Morley AA (1985) Measurement of micronuclei in lymphocytes. *Mutat Res* **147**: 29–36

Geard CR and Chen CY (1990) Micronuclei and clonogenicity following low- and high-dose-rate irradiation of normal human fibroblasts. *Radiat Res* **124**: S56–S61

Grote SJ, Joshi GP, Revell SH and Shaw CA (1981) Observations of radiation-induced chromosome fragment loss in live mammalian cells in culture, and its effect on colony-forming ability. *Int J Radiat Biol* **39**: 395–408

Hunter RD, Cowie VJ, Blair V and Cole MP (1986) A clinical trial of two conceptually different radical radiotherapy treatments in stage III carcinoma of the cervix. *Br J Cancer* **37**: 23–27

Johansen J, Bentzen SM, Overgaard J and Overgaard M (1996) Relationship between the in vitro radiosensitivity of skin fibroblasts and the expression of subcutaneous fibrosis, telangiectasia, and skin erythema after radiotherapy. *Radiother Oncol* **40**: 101–109

Kiltie AE, Orton CJ, Ryan A, Roberts SA, Marples B, Davidson SE, Hunter RD, Margison GP, West CML and Hendry JH (1997) A correlation between residual DNA double strand breaks and clonogenic measurements of radiosensitivity in fibroblasts from pre-radiotherapy cervix cancer patients. *Int J Radiat Oncol Biol Phys* **39**: 1137–1144

Knight RD, Parshad R, Price FM, Tarone RE and Sanford KK (1993) X-ray-induced chromatid damage in relation to DNA repair and cancer incidence in family members. *Int J Cancer* **54**: 589–593

Odagiri Y, Takemoto K and Fenech M (1994) Micronucleus induction in cytokinesis-blocked bone marrow cells in vitro following in vivo exposure to x-irradiation and cyclophosphamide. *Environ Mol Mutag* **24**: 61–67

Parshad R, Price FM, Bohr VA, Cowans KH, Zujewski JA and Sanford-KK (1996) Deficient DNA repair capacity, a predisposing factor in breast cancer. *Br J Cancer* **74**: 1–5

Russell NS, Knaken H, Bruinvis IAD, Hart AAM, Begg AC and Lebesque JV (1994) Quantification of patient to patient variation of skin erythema developing as a response to radiotherapy. *Radioth Oncol* **30**: 213–221

Savage JR (1988) A comment on the quantitative relationship between micronuclei and chromosomal aberrations. *Mutat Res* **207**: 33–36

Scott D and Highway J (1986) Identification of individuals at risk: cytogenetic and molecular methods. *Prog Clin Biol Res* **209B**: 205–212

Scott D, Spreadborough A, Levine E and Roberts SA (1994) Genetic predisposition in breast cancer. *Lancet* **344**: 1444

Scott D, Barber JBP, Levine EL, Burrill W and Roberts SA (1998) Radiation-induced micronucleus induction in lymphocytes identifies a high frequency of radiosensitive cases among breast cancer patients: a test for predisposition? *Br J Cancer* **77**: 614–620

West CML (1995) Intrinsic radiosensitivity as a predictor of patient response to radiotherapy. *Br J Radiol* **68**: 827–837

Williams KJ, Boyle JM, Birch JM, Norton JD and Scott D (1997) Cell cycle arrest defect in Li–Fraumeni syndrome: a mechanism of cancer predisposition? *Oncogene* **14**: 277–282

© Cancer Research Campaign 1998